# Assessment of Hemodynamics in a Rat Model of Liver Cirrhosis with Precancerous Lesions Using Multislice Spiral CT Perfusion Imaging

**DOI:** 10.1155/2013/813174

**Published:** 2013-06-20

**Authors:** Guolin Ma, Rongjie Bai, Huijie Jiang, Xuejia Hao, Zaisheng Ling, Kefeng Li

**Affiliations:** ^1^Department of Radiology, China-Japan Friendship Hospital, Beijing 100029, China; ^2^School of Medicine, University of California, San Diego (UCSD), San Diego, CA 92093, USA; ^3^Department of Radiology, Beijing Jishuitan Hospital, Beijing 100035, China; ^4^Department of Radiology, Second Affiliated Hospital, Harbin Medical University, Harbin 150086, China

## Abstract

*Rationale and Objectives*. To develop an optimal scanning protocol for multislice spiral CT perfusion (CTP) imaging to evaluate hemodynamic changes in liver cirrhosis with diethylnitrosamine- (DEN-) induced precancerous lesions. *Materials and Methods*. Male Wistar rats were randomly divided into the control group (*n* = 80) and the precancerous liver cirrhosis group (*n* = 40). The control group received saline injection and the liver cirrhosis group received 50 mg/kg DEN *i.p.* twice a week for 12 weeks. All animals underwent plain CT scanning, CTP, and contrast-enhanced CT scanning. Scanning parameters were optimized by adjusting the diatrizoate concentration, the flow rate, and the delivery time. The hemodynamics of both groups was further compared using optimized multislice spiral CTP imaging. *Results*. High-quality CTP images were obtained with following parameters: 150 kV; 150 mAs; 5 mm thickness, 5 mm interval; pitch, 1; matrix, 512 × 512; and FOV, 9.6 cm. Compared to the control group, the liver cirrhosis group had a significantly increased value of the hepatic arterial fraction and the hepatic artery perfusion (*P* < 0.05) but significantly decreased hepatic portal perfusion and mean transit time (*P* < 0.05). *Conclusion*. Multislice spiral CTP imaging can be used to evaluate the hemodynamic changes in the rat model of liver cirrhosis with precancerous lesions.

## 1. Introduction

Hepatocellular carcinoma (HCC) is the most common type of primary liver cancer that poses a serious threat to human health [1]. Early detection of pathological changes before the precancerous lesions occur is critical for the treatment and prognosis of the disease. Diethylnitrosamine- (DEN-) induced HCC in rat is an experimental model that mimics the pathological process of human HCC [2–4]. This model has a relatively simple procedure and a high induction rate. It has been widely used for the study of HCC and precancerous lesions of liver cirrhosis, particularly for the image analysis of the progression of HCC [5, 6].

The development of HCC has been known to be accompanied by gradual changes in hepatic arterial and portal hemodynamics. The HCC initiates from the dysplastic nodule (DN) in cirrhotic livers and progresses through multiple levels, including low-grade DN, atypical adenomatous hyperplasia, DN containing foci of HCC subnodules, typical small HCC, and advanced HCC [7]. DN can be classified as low grade and high grade based on the pathological features. The DN progression is accompanied by hemodynamic changes in the hepatic artery and portal vein. The blood supply decreases in the portal vein while increases in the hepatic artery, leading to precancerous lesions of liver cirrhosis. Since the occurrence of precancerous lesions is closely related to the changes in the blood flow, accurate assessment of hemodynamics during the development of precancerous lesions is extremely critical for the early detection of cirrhotic liver.

CT perfusion (CTP) is a noninvasive functional imaging method for the assessment of tissue and organ perfusions [8, 9]. CTP can measure even capillary hemodynamics due to its high temporal and spatial resolutions and good repeatability; therefore, CTP has been transformed from a morphological diagnosis tool to a functional diagnosis one. CTP has now been widely used in the clinics for quantitative evaluation of liver cirrhosis and hemodynamic changes of HCC. Recent studies showed that 16-slice spiral CTP imaging significantly shortened the scan time and improved the density resolution of the image and seven perfusion parameters were calculated through the mathematical model of deconvolution analysis [10, 11]. Although development of quantitative diagnostic methods has improved the diagnostic accuracy of HCC and precancerous lesions [12], some technical issues still need to be addressed. Especially, when CTP is applied to the animal model, small body size limits the quality of the images. The aim of this study was to develop an optimal imaging protocol to acquire high-quality multislice spiral CT perfusion images in the rat model of liver cirrhosis. Our results suggested that the enhancement time adjustment can be used to improve the diagnostic accuracy of multislice spiral CT in rats. In addition, hemodynamic changes in rat livers with precancerous lesions can be monitored using CTP.

## 2. Materials and Methods

### 2.1. Animal and Experimental Groups

One hundred and twenty male Wistar rats (two-month old, body weight ranged from 180 to 200 g) were housed with regular diet for two weeks before the experiment. The rats were then divided into two groups: (1) the control group (*n* = 80) received saline injection; and (2) the precancerous liver cirrhosis group (*n* = 40) received 50 mg/kg DEN (0.95 g/mL with 99.9% purity purchased from Sigma, St. Louise, MO, USA) via intraperitoneal injection twice a week for 12 weeks before the examination. Animals were observed daily during the period of treatment for the sign of stress such as decreased activity, reduced water and food consumption, or ruffled fur. If the animals developed any sign of abnormality, the DEN treatment was delayed or terminated. All animal protocols were approved by the animal ethics committee of Harbin Medical University.

### 2.2. Multislice CT Imaging Examination

The rat livers in the control group underwent plain CT scan and perfusion scan with multislice spiral CT (LightSpeed 16-slice spiral CT; GE Healthcare, Milwaukee, WI, USA). Before the CT scan, the diatrizoate was given intravenously using a high-pressure syringe into the tail vein. Plain CT scan conditions were as follows: 5.0 mm slice thickness, 5.0 mm interval, 1.0 pitch, 120 kV tube voltage, 60 mA tube current, 512 × 512 matrix, FOV 9.6 cm, and 50 s scanning [13]. After the plain CT scan, a slice with a clear image of the portal vein and the abdominal aorta was selected for CTP. To optimize the imaging condition, variables including contrast agent concentrations (19%, 38%, 57%, or 76%), injection rates (0.3 or 0.5 mL/s), and injection time (1 s, 2-3 s, 4-5 s, or 6 s) were used in different combinations with 4 rats for each one (Table 1). The best scanning condition was determined by comparing the image quality of the CTP. The CTP acquisition parameters were determined as follows: 150 kV; 150 mAs; slice thickness 5 mm; interval, 5 mm; pitch, 1; matrix, 512 × 512; and FOV, 9.6 cm. The time-density curves (TDCs) of artery and portal vein were obtained during 50 seconds of scanning.

### 2.3. Image Evaluation and Data Analysis

Acquired data were transferred to the GE AW4.2 workstation and Perfusion 2 software (a deconvolution algorithm) was used for data processing. The TDCs and the pseudo-color functional map were obtained. Various perfusion parameters of the liver tissue were measured in the region of interest (ROI). The deconvolution algorithm allowed calculation of seven parameters for each ROI, including the hepatic blood flow (HBF, mL/min/100 mg), the hepatic blood volume (HBV, mL/100 mg), the mean transit time (MTT, s) of the contrast agent, the capillary permeability-surface area product (PS, mL/min/100 mg), the hepatic arterial fraction (HAF, %), the hepatic artery perfusion (HAP, mL/min/100 mg), and the hepatic portal perfusion (HPP, mL/min/100 mg). The HBF, HAP, and HPP were calculated using the following equations (1), (2), and (3), respectively,

(1) HBF=HAP+HPP,

(2) HAP=HBF×HAF,

(3) HPP=HBF×(1−HAF).

Three ROIs were carefully demarcated to obtain satisfactory TDCs, including the portal vein, abdominal aorta, and liver parenchyma. The abdominal aorta was used instead of the hepatic artery because the hepatic artery was too thin. All ROIs were distanced from the edge of the liver to avoid the influence of partial volume effects. Large branches of the blood vessels in the liver were also avoided. Enhanced CT was performed 10 minutes after the perfusion scanning was completed. Three phases of enhancement were summarized as follows based on the TDC of perfusion imaging: the hepatic arterial phase (7 s), the hepatic portal venous phase (15 s), and the delayed phase (23–31 s).

### 2.4. Histological Examination

After imaging, the rats were sacrificed and the livers were fixed in 10% formaldehyde and embedded in paraffin for immunohistochemical study. Consecutive 4 *μ*m cryostat sections were obtained, mounted on glass slides, and stained for histological evaluation using standard haematoxylin and eosin (HE) staining.

### 2.5. Statistical Analysis

All data were expressed as mean ± SD. All statistical analyses were carried out using SPSS version 11.5 (SPSS Inc., Chicago, IL, USA). A two-tailed Student's *t*-test was used to determine differences in perfusion parameters between groups. Changes in the different perfusion parameters during tumor growth were evaluated by one-way analysis of variance (ANOVA). A *P* value of <0.05 was considered to be statistically significant.

## 3. Results

### 3.1. Establishment of the Rat Model of Precancerous Liver Cirrhosis

Rats in the control group (*n* = 80) were active and gained weight during the experimental period and no death occurred. Rats in the liver cirrhosis group showed decreased activities after 4-week DEN treatment, accompanied by the occurrence of gray furs and reduced food intake. Most rats in the DEN-treated group showed a significant weight loss. Twenty rats died before the completion of the 12-week treatment of DEN. The main causes of the early death were DEN-induced toxicity and pulmonary infection, while the main cause of death at the later period of the treatment was the rupture of hepatic nodules or the infection in cirrhotic livers. Five rats died after anesthesia before CT scanning.

### 3.2. Modification of the CTP Scanning Protocol

The permutations of different factors including contrast agent concentrations (19%, 38%, 57%, and 76%), injection rate (0.3 and 0.5 mL/s), and injection time (1 s, 2-3 s, 4-5 s, and 6 s) were compared in the control group. Initially, a total of 20 conditions were tested and some conditions were combined as the same group when the same imaging effects were observed after analysis. Finally, 13 imaging conditions were compared in this study and results were summarized in Table 1. The contrast agent of 19% and 38% concentrations showed similar low and flat TDC and no pseudo-color map was generated. When the concentration of contrast agent was increased to 76%, it was difficult to perform the injection due to the high viscosity. Injection time at 1 s, 2-3 s, and 4-5 s failed to generate pseudo-color map, while injection time at 6 s generated satisfactory pseudo-color image. The injection at the rate of 0.3 mL/s failed to generate image. After comparing all the imaging conditions, we found that tail vein administration of 57% contrast agent at a flow rate of 0.5 mL/s for 6 s could yield a satisfactory TDC that well reflected the dynamics of the hepatic artery and portal vein (Table 1). In the pseudo-colored images, the liver parenchyma with an elevated blood flow showed an increase in perfusion detected by CPT.

### 3.3. Adjustment of CT Enhancement Time

**Figure 1:**
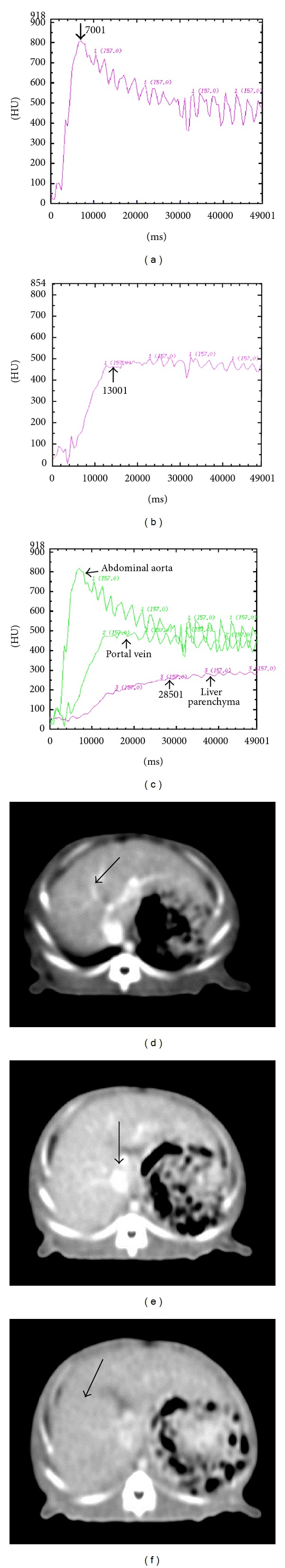
The TDCs obtained during perfusion imaging. (a) The TDC of the abdominal aorta showed a rapid increase and the time to peak was 7.2 ± 3.6 s. (b) The TDC of the portal vein showed a slow increase and the time to peak was 14.7 ± 4.2 s. (c) The TDC of the liver parenchyma showed a slow increase and the time to peak was 27.0 ± 4.5 s. (d) The hepatic arterial phase. The abdominal aorta showed obvious enhancement, while the portal vein was not clearly displayed. (e) The hepatic portal venous phase. The trunk of the portal vein and the left branch showed obvious enhancement. (f) The delayed phase. The liver parenchyma showed obvious homogeneous enhancement, while enhancement of the abdominal aorta and portal vein decreased.

Representative TDCs for the abdominal aorta, portal vein, and liver parenchyma in the control group are presented in Figures 1(a)–1(c). The results of enhanced CT scanning images were evaluated according to the characteristics of liver enhancement at each phase. In this study, TDCs showed an arterial and portal enhancement peak at 7.2 ± 3.6 s and 14.7 ± 4.2 s, respectively, followed by the stationary phase of parenchyma enhancement at 27.0 ± 4.5 s. The three phases of the contrast-enhanced CT were hepatic arterial phase (7 s), hepatic portal venous phase (15 s), and the delayed phase (23–31 s). The TDC of the abdominal aorta had a rapid ascent with a peak value of 800 HU at 7001 ms (Figure 1(a)). For the portal vein, the TDC had a rapid ascent and slow descent. Compared to the aorta, the time to the peak value was longer (14.7 ± 4.2 s) and the amplitude of the TDC was smaller (Figure 1(b)). The TDC of the liver parenchyma had a slow rise in the arterial phase and a rapid rise in the portal venous phase. The time to the plateau was 27.0 ± 4.5 s (Figure 1(c)). No abnormal perfusion areas were observed in the liver parenchyma of the control group. Based on the above TDC results, the time of the enhanced scan was readjusted to 7 s for the hepatic arterial phase (Figure 1(d)), 15 s for the hepatic portal venous phase (Figure 1(e)), and 23–31 s for the delayed phase (Figure 1(f)). The CT images after the adjustment displayed the enhancement characteristics of the liver parenchyma, hepatic artery, portal vein, and inferior vena cava in all of the phases.

### 3.4. Comparison of Pathological Changes in Normal and Cirrhotic Livers in a Rat

**Figure 2:**
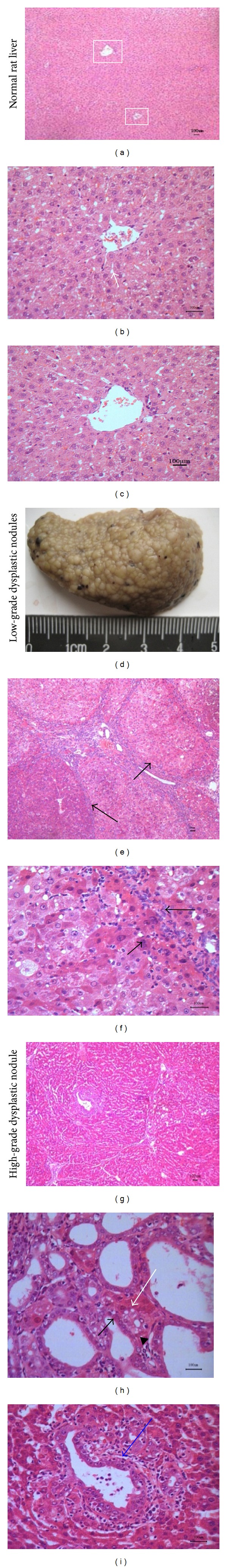
The pathological images of normal and cirrhotic rat livers. (a) Tissue from normal rat livers. (b)-(c) Images of normal tissue with a higher magnification. Images showed typical lobular architecture and uniform distribution of liver cells. (d) Specimen of liver sample with low-grade DN. The liver showed gray surface and many nodules with various sizes. (e) Low-grade DN in a cirrhotic liver. (f) Image of low-grade DN with a higher magnification. Images showed increased number of cells with higher nuclei-to-cytoplasm ratio (black arrows). (g) High-grade DN in a cirrhotic liver. (h)-(i) Image of high-grade DN with a higher magnification. The reticular structure of nodule was reduced. Steatosis (arrow head) and clustered Mallory bodies (white arrow in (h)) of various sizes are found in the nodules (white arrow). Isolated adenoid structure was also found (arrow in (i)). Arrows indicated the ROI regions. Scale bar = 100 *μ*M in (a) to (c) and (e) to (i).

The normal rat livers showed typical lobular architecture and uniform distribution of liver cells visualized with HE staining (Figures 2(a)–2(c)). Cirrhotic livers with low-grade DN (Figure 2(d)) displayed gray surface and nodules with various sizes. The hepatocyte plates reached up to three-cell thick (black arrows in Figures 2(e) and 2(f)). Cirrhotic livers with high-grade DN (Figures 2(g)–2(i)) exhibited reduced reticular structure of nodule. Steatosis (arrow head in Figure 2(h)) and clustered Mallory bodies (white arrow) of various sizes were also found in the nodules. Isolated adenoid structure was also found (arrow in Figure 2(i)).

### 3.5. Comparison of CTP Images of Normal and Cirrhotic Livers with Precancerous Lesions

**Figure 3:**
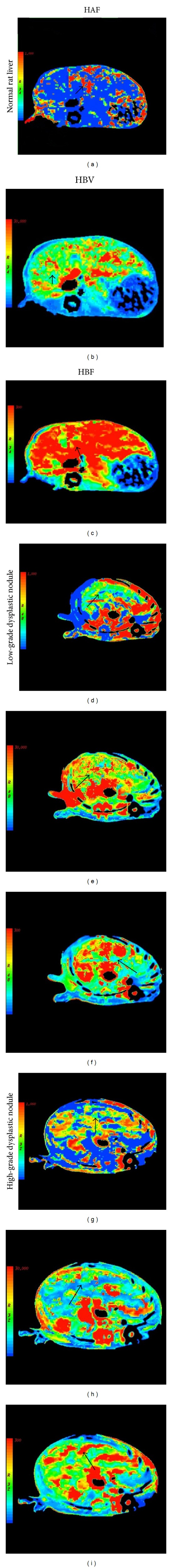
The pseudo-colored perfusion images of HAF, HBV, and HBF of liver parenchyma. (a)–(c) Normal livers. (d)–(f) Low-grade DN in the cirrhotic liver. (g)–(i) High-grade DN in the cirrhotic liver. Measurement values from high to low were indicated from red to blue.

Representative pseudo-colored perfusion images of HAF, HBV, and HBF for normal liver parenchyma (Figures 3(a)–3(c)), cirrhotic livers with low-grade DN (Figures 3(d)–3(f)) and high-grade DN (Figures 3(g)–3(i)) were presented. Compared to the normal livers, cirrhotic livers with precancerous nodules had significantly higher values of HAF (0.63 ± 0.10 versus 0.25 ± 0.09, *P* < 0.05) and HAP (217.74 ± 124.32 versus 107.12 ± 72.63, *P* < 0.05) but decreased MTT (2.27 ± 1.23 versus 9.34 ± 16.99, *P* < 0.05) and HPP (137.24 ± 101.61 versus 326.60 ± 171.69, *P* < 0.05) (Table 2). Compared to the control group, liver parenchyma with precancerous nodules showed a trend of decreased HBF and HBV and increased PS, while no significant difference was detected between the two groups.

## 4. Discussion

In this study, by using a rat model of liver cirrhosis with DEN-induced precancerous lesions, we developed a protocol to obtain high-quality images using multislice spiral CTP. The enhancement time for each phase was adjusted according to the peak timing in TDCs of normal liver CTP. Satisfactory images were obtained by adjusting the scanning time of three-phase contrast-enhanced CT and optimizing administration methods for the contrast agent. Moreover, by using this protocol we compared the hemodynamics between normal and cirrhotic livers with precancerous lesions. Our results might improve the value of multislice spiral CTP imaging in the study of using animal model of liver cirrhosis with precancerous lesions.

Commonly used imaging methods for the study of the hemodynamics in cirrhotic livers include sorbitol clearance rate, radio-isotope scanning, ultrasound-liver perfusion, electromagnetic flow meter, and perfusion MR imaging [14–16]. Measurement of liver perfusion using sorbitol clearance is affected by the level of liver enzymes and transporters; therefore, it is not suitable for patients with a severe liver disease or significant changes in liver enzymes [14]. Isotope scanning (single photon emission computed tomography and positron emission tomography), a commonly used functional imaging method, has an advantage in detecting the perfusion features in a large area of hepatic artery and portal vein [17, 18]. However, it can neither distinguish the perfusion patterns of the dual blood supply in the liver nor calculate the absolute value of perfusion per volume unit accurately [19]. The application of isotope scanning is also limited by its low spatial resolution, labeling, and detection efficiency. Although ultrasound provides real-time and rapid measurements of liver perfusion and can measure the blood flow in large vessels of hepatic parenchyma, it has difficulty in measuring hepatic microcirculation. Compared to all above techniques, CTP imaging has a great application potential due to its advantage of high resolution in the soft tissue and that no radiation is needed for the process. However, currently there is no standard scanning procedure for the CTP imaging. CTP imaging is susceptible to chemical shift and has a limited time resolution. Furthermore, the nonlinear relationship between the amount of contrast agent and the signal strength has made it difficult to accurately determine the hemodynamics of hepatic artery and portal vein among different types of DN [20]. In this study, we determined the desirable dosage and concentration of the contrast agents for CPT imaging in rat liver. Our results will be useful for the application of CPT imaging in the study of hepatic hemodynamics in the animal model of liver cirrhosis [21].

### 4.1. Establishment of Rat Model of Liver Cirrhosis Using DEN-Induced Precancerous Lesions

Currently, three rat models of HCC have been reported. The transgenic HCC model is mainly used to study the signaling pathways during the development of HCC [22]. In the xenograft HCC model, the tumor is inoculated in the normal liver. Although the xenograft model is easy to establish and has a high success rate, the HCC is significantly different from the surrounding tissues and the disease progression differs from that in most clinical HCC cases [23]. Another commonly used model is chemical (DEN-) induced HCC in which the progression of disease from liver damage to cirrhosis and malignant HCC can be controlled by the DEN dosage. In this study, the DEN-induced HCC in rats was used as an experimental animal model. In our experiment, we observed a significant weight loss caused by DEN toxicity in most DEN-treated rats. Twenty out of forty rats died before the completion of 12-week DEN treatment and 5 rats died after anesthesia, suggesting a high mortality rate in the DEN-induced HCC model. This observation is consistent with previous reports about the DEN-induced HCC model [24, 25]. The rat model of precancerous lesions is established on the basis of liver cirrhosis, a stage of which can be controlled by the dosage of DEN. DEN dosage lower than 10 mg/kg can only initiate cancerous transformation, while 10 mg/kg DEN can lead to liver fibrosis [26]. DEN dosage higher than 25–30 mg/kg can promote the tumor progression [27]. Park et al. reported that rats receiving intraperitoneal injection of 50 mg/kg DEN twice a week had HCC developed from the promotion stage to the progression stage after 12-week DEN treatment [28]. In this study, we used the dosage of 50 mg/kg DEN which greatly shortened the time for model establishment and effectively controlled the progress of early HCC development (liver cirrhosis and HCC). Our results indicate that intraperitoneal injection of 50 mg/kg DEN twice a week for 12 weeks in rats can establish a useful animal model of HCC for the study of hepatic hemodynamics using CTP imaging, and high quality CTP images of cirrhotic livers can be obtained.

### 4.2. Technical Improvement of CTP Imaging in Rats

The Glisson ductal system, hepatic vein, and artery in Wistar rats are similar to those in humans [29]. Although the rat is an ideal animal model for the study of hepatic hemodynamics, the small body of rat and improper administration of contrast agent may result in low-quality CPT images. Therefore, we adjusted the concentration and dosage of the contrast agent and the flow rate to achieve high-quality images. Good TDCs of abdominal artery and portal vein are critical for CTP imaging. The mathematical models used for TDCs include nondeconvolution and deconvolution and the latter one was used in this study. The venous outflow of iodinated contrast agent was calculated through the surplus function of the deconvolution model to incorporate the arterial inflow and venous outflow of liver perfusion. Therefore, the perfusion parameters and the pseudo-colored images generated using this method could more directly reflect the real status of hemodynamics inside the lesions. After the comparison of different imaging conditions, we found that administration of 57% contrast agent at a flow rate of 0.5 mL/s for 6 s could yield a TDC that well reflected the changes in the hepatic artery and portal vein. This experimental procedure had a high maneuverability and repeatability, allowing accurate analysis of perfusion parameters.

In this study, we administered the contrast agent using a high-pressure syringe to achieve a rapid injection. This protocol allowed us to start the injection of contrast agent and scanning simultaneously. Therefore, the start point of the X coordinate in the TDC is around 0, which is different from the 3–5 s delay reported previously [13, 30]. In previous studies, the agent was injected manually 3–5 s after the scanning started. By using a high-pressure syringe, we could obtain a complete TDC and more accurate perfusion parameters.

In this study, the HAF of normal rat livers showed a range of 16%–34%. Although the literatures have reported that types of agent used for CTP procedure and injection path (through tail vein or jugular vein) might affect HAF [31, 32], it has been accepted that the HAF in normal rat livers is about 20% [33]. Therefore, the HAF reported in this study is within the normal range and in line with the widely accepted 1 : 3-4 ratio of blood flow in the hepatic artery and portal vein. This suggests that the perfusion parameters used in our experiment well reflected the changes in the blood flow in normal rat livers.

### 4.3. Adjustment of CT Enhancement Phase Based on TDCs of Liver CTP Imaging

The TDC is a point-to-point changing curve of CT value over a continuous time period, which could reflect the enhancement level of the tissue directly. Our TDCs results reflected the characteristics of the three-phase contrast-enhanced CT images and all images clearly showed the structure of liver parenchyma and hepatic vessels. The peak time of TDCs reported in our study differed from the previous report [34] which showed an arterial enhancement peak at 6 s, 9 s, or 10 s, and a portal enhancement peak at 12 s, 15 s, or 18 s. The TDCs from 8 rats in our study showed an arterial enhancement peak at 2 s. However, the start points of the curves were at 200 HU–500 HU in the previous study. This is probably due to the delayed start of the scanning. It has been reported that increased flow rate of the contrast agent can significantly shorten the peak time and increase the peak value of the arterial enhancement [35], which is consistent with our result. When the flow rate was 0.3 mL/s, the TDC peak of abdominal artery was mostly below 200 HU and the peak time was 12–15 s. When the flow rate was increased to 0.5 mL/s, the TDC peak of abdominal artery was mostly above 600 HU and the peak time was 7–10 s.

### 4.4. Evaluation of Hemodynamic Change in Cirrhotic Liver Using CTP Imaging

In normal livers, the hepatic artery accounts for 20–25% and the portal vein for 75–80% of blood supply. We reported the HAF value at approximately 20% in normal rat livers (i.e., HAP : HPP = 1 : 4), indicating that CTP well reflected the hemodynamics of livers under normal physiological conditions. In normal livers, the stability of liver blood circulation is maintained through the equilibrium between the HAP and HPP, while this stability is disrupted during the development of precancerous lesions or liver cirrhosis. In this study, we observed increased HAP and decreased HPP in the precancerous lesions group. This is similar to the result reported by Tsushima et al. [36], in which the rise of HAP compensates the decrease in HPP and the increase in HAP is often less than the decrease in HPP. 

The development of liver cirrhosis is a gradual process. It can be reversed at the early stage but hardly at the later stage. The hemodynamic change occurs before a significant morphological change and can be detected during the early stage of liver cirrhosis. The histopathological changes, including hepatic cell transformation, necrosis, regenerative nodules, hyperplasia of fibrous connective tissue, and pseudo-lobule formation, can cause not only structural abnormality in livers and but also a decrease in the area of the intrahepatic vascular bed that is associated with increased perfusion resistance and abnormal perfusion. With the progression of disease, the portal blood supply decreases while the arterial blood supply increases. HAF reflects the change in the ratio of hepatic arterial-to-portal venous blood flow in liver lesions [13, 30, 37]. As we reported in the previous study [38], both HAP and HAF increased in malignant liver tumors. In the normal rat liver, the terminal branches of hepatic artery and portal vein are connected in the liver sinusoid, in which endothelial cells have unique anatomical structures. Abundant discontinuous basement membrane pores with a diameter of 50–200 nm allow intensive interaction between liver cells and blood. Liver lesions, particularly the formation of pseudo-lobules and hyperplasia of fibrous tissues, can cause structural changes in the liver sinusoid and lead to hemodynamic changes in hepatic microcirculation and reduced portal vein perfusion. These changes might be caused by increased pressure in the liver sinusoid due to hyperplasia of fibrous tissues and significantly reduced pore structure in the nonbasement membrane among the sinusoidal endothelial cells. The sinusoidal capillarization and accumulation of collagen between the sinusoids and hepatocytes might also contribute to a decrease in portal perfusion.

### 4.5. Limitations of This Study

In this study, the CTP scanning protocol was determined on normal rats livers. However, livers with cirrhosis or precancerous lesions have altered characteristics of circulation and vascular structure. This might affect the enhancement timing used for the CTP scanning program in cirrhotic livers. Additionally, our protocol can only detect the hemodynamic changes in the entire liver due to the small body of the rat and the limited resolution of 16-slice spiral CT. 

## 5. Conclusion

In this study, we optimized CTP scanning parameters to evaluate the hemodynamic changes in cirrhotic rat livers more accurately. Our procedure provides an effective diagnostic imaging method to measure the blood flow changes in the hepatic artery and portal vein of small animals. To improve the application of liver CTP in the diagnosis of livers precancerous lesions or cirrhosis, the correlation between hemodynamics obtained with CTP imaging and pathological changes in livers still needs further investigation.

## Figures and Tables

**Table 1:** Combinations of imaging conditions.

Injection time (s)	Concentration (%)	Injection rate (mL/s)	Effects and analysis of perfusion imaging	Methods to improve image quality
1	19–76	0.3–0.5	Low concentration, short duration, and no pseudo-color map	Extend the injection time
1	76	0.5	High concentration, slow injection rate, and no pseudo-color map	Unfeasible method
2~3	76	0.3	Short duration and no pseudo-color map	Extend the injection time
2~3	19–57	0.3–0.5	Low concentration, short duration, and no pseudo-color map	Extend the injection time
2~3	76	0.5	High concentration, slow injection, and no pseudo-color map	Unfeasible method
4~5	76	0.3	Short duration and no pseudo-color map	Extend the injection time
4~5	19–38	0.3–0.5	Low concentration, short duration, and no pseudo-color map	Extend the injection time
4~5	57	0.5	Poor quality of pseudo-color map	Extend the injection time
4~5	76	0.5	High concentration, slow injection, and no pseudo-color map	Unfeasible method
6	76	0.3	Poor quality of pseudo-color map	Decrease concentration
6	19–38	0.3–0.5	Low concentration and no pseudo-color map	Increase concentration
**6**	**57**	**0.5**	**Good pseudo-color map**	**Feasible method**
6	76	0.5	High concentration, slow injection, and no pseudo-color map	Unfeasible method

Bold: the optimal imaging protocol we used in this study.

**Table 2:** Values of CTP imaging in the normal and cirrhotic rat livers.

ROI	Normal livers (80)	Precancerous livers (40)	*P* value
HAF (%)	0.25 ± 0.09	0.63 ± 0.10*	0.00001
HBF (mL/min/100 mg)	433.72 ± 225.32	354.99 ± 211.60	0.095
HBV (mL/100 mg)	40.09 ± 94.03	12.43 ± 6.30	0.073
MTT (s)	9.34 ± 16.99	2.27 ± 1.23*	0.012
PS (mL/min/100 mg)	36.68 ± 31.34	45.36 ± 20.35	0.132
HAP (mL/min/100 mg)	107.12 ± 72.63	217.74 ± 124.32*	0.00002
HPP (mL/min/100 mg)	326.60 ± 171.69	137.24 ± 101.61*	0.00001

Notes: All parameters were measured three times. *P < 0.05 compared with the control group.
